# Laser-Induced Graphene Decorated with MOF-Derived NiCo-LDH for Highly Sensitive Non-Enzymatic Glucose Sensor

**DOI:** 10.3390/molecules29235662

**Published:** 2024-11-29

**Authors:** Longxiao Li, Yufei Han, Yuzhe Zhang, Weijia Wu, Wei Du, Guojun Wen, Siyi Cheng

**Affiliations:** School of Mechanical Engineering and Electronic Information, China University of Geosciences, Wuhan 430074, China; lilongxiao@cug.edu.cn (L.L.)

**Keywords:** laser-induced graphene, NiCo-LDH, flexible, ZIF-67, glucose sensor

## Abstract

Designing and fabricating a highly sensitive non-enzymatic glucose sensor is crucial for the early detection and management of diabetes. Meanwhile, the development of innovative electrode substrates has become a key focus for addressing the growing demand for constructing flexible sensors. Here, a simple one-step laser engraving method is applied for preparing laser-induced graphene (LIG) on polyimide (PI) film, which serves as the sensor substrate. NiCo-layered double hydroxides (NiCo-LDH) are synthesized on LIG as a precursor, utilizing the zeolitic imidazolate framework (ZIF-67), and then reacted with Ni(NO_3_)_2_ via solvent-thermal methods. The sensitivity of the non-enzymatic electrochemical glucose sensor is significantly improved by employing NiCo-LDH/LIG as the sensing material. The porous and interconnected structure of NiCo-LDH, derived from ZIF-67, enhances the accessibility of electrochemically active sites, while the incorporation of LIG ensures exceptional conductivity. The combination of NiCo-LDH with LIG enables efficient electron transport, leading to an increased electrochemically active surface area and enhanced catalytic efficiency. The fabricated electrode achieves a low glucose detection limit of 0.437 μM and demonstrates a high sensitivity of 1141.2 and 631.1 μA mM^−2^ cm^−2^ within the linear ranges of 0–770 μM and 770–1970 μM, respectively. Furthermore, the NiCo-LDH/LIG glucose sensor demonstrates superior reliability and little impact from other substances. A flexible integrated LIG-based non-enzymatic glucose sensor has been developed, demonstrating high sensitivity and suggesting a promising application for LIG-based chemical sensors.

## 1. Introduction

In recent years, the number of diabetes patients worldwide has been steadily increasing, leading to millions of deaths each year, which has drawn greater attention to the early prevention and diagnosis of diabetes [1]. Monitoring and controlling blood glucose levels in the early stages of diabetes is critical, which has driven the development of electrochemical glucose sensors with high sensitivity, stability, and low cost. Electrochemical glucose sensors can be classified into enzyme-based and non-enzyme-based types [2]. Enzyme-based sensors are susceptible to external factors such as temperature and pH, leading to instability [3], while non-enzyme-based sensors offer advantages such as high sensitivity, stability, rapid response, and low cost, making them more suitable for practical applications.

Electrode materials are crucial in determining the performance of sensors, with graphene being widely utilized across various fields due to its exceptional carrier mobility, large specific surface area, and remarkable mechanical properties [4,5]. These characteristics make graphene highly suitable for applications such as field-effect transistors [6,7,8,9], transparent electrodes [10,11,12], energy storage devices [13,14,15,16], and electrochemical sensors [17,18,19]. However, conventional graphene fabrication methods, such as mechanical exfoliation, oxidation-reduction techniques, and chemical vapor deposition, are often costly and complex [20,21,22]. In contrast, Tour’s research group developed a more efficient and cost-effective technique by irradiating polyimide (PI) films with lasers to produce laser-induced graphene (LIG). This process enables the rapid production of graphene electrodes, which is particularly beneficial for the manufacturing of wearable devices [23,24,25].

Moreover, electrode materials are critical in electrochemical catalytic oxidation reactions and vital for electrochemical sensors. Metal–organic frameworks (MOFs) and layered double hydroxides (LDHs) have emerged as promising electrode materials due to their excellent catalytic performance and tunable structures. MOFs are crystalline materials composed of two- or three-dimensional infinite lattices formed by metal cations coordinated with multidentate organic ligands [26]. They are characterized by a large surface area, adjustable pore structures, and abundant open, active sites that promote electrolyte penetration [27,28]. Zeolitic imidazolate frameworks (ZIFs), a subclass of MOFs, are widely used in sensors, particularly cobalt-based MOFs (Co-MOFs) [29]. However, MOFs suffer from poor conductivity and limited stability [30], which can be addressed by converting them into layered double hydroxides, such as NiCo-LDH and MoCo-LDH [31].

Conductivity enhancements, densification of active sites, and larger active surface areas can greatly improve electrode materials’ electrochemical performances [32]. With the bimetal composition and porous structure derived from their precursors, LDHs are widely used in electrochemical systems because of their exceptional conductivity, high concentration of active sites, and large particular areas of surface [33,34,35]. These characteristics render LDHs well-suited for use in applications, including supercapacitor electrodes and non-enzymatic glucose sensors, where a large surface area, exceptional mechanical stability, and adjustable thermal conductivity are of utmost importance [36]. Among the different types of LDHs, NiCo-LDH possesses various oxidation states of nickel and cobalt, which can generate numerous electroactive centers and substantially enhance both conductivity and electrocatalytic efficiency [37,38]. Numerous studies have confirmed the strong electrochemical catalytic activity of NiCo-LDH, particularly for glucose sensing [39,40].

To date, numerous glucose sensors based on LIG or NiCo-LDH have been developed and applied, underscoring the significant potential of these materials in glucose detection [41,42,43,44,45]. However, most non-enzymatic glucose sensors utilizing LIG typically employ transition metal elements and their oxides as the catalytic materials [46,47,48,49,50]. Research focusing on the integration of LIG with metal–organic framework (MOF)-derived NiCo-LDH remains relatively scarce. The combination of LIG’s high conductivity and large specific surface area with the exceptional catalytic properties of MOF-derived NiCo-LDH offers considerable promise. Despite this potential, the exploration of this novel composite material is still in its nascent stages, and further investigation is needed to fully realize its advantages and capabilities in glucose sensing.

This study presents the development of a novel NiCo-LDH synthesized from MOF on LIG. An in-depth analysis of the engraving process achieved through the optimization of laser parameters is provided. The subsequent characterization of the NiCo-LDH/LIG composite focuses on its morphology, structural features, and electrochemical properties. Additionally, this study offers a detailed discussion of the structural advantages and electrochemical performance of the NiCo-LDH/LIG composite. Electrochemical assessments revealed that the NiCo-LDH electrode demonstrates exceptional sensitivity, selectivity, and a broad detection range. These attributes underscore the significant potential of this electrode for applications in glucose sensing.

## 2. Results and Discussion

### 2.1. Fabrication and Performance Optimization of LIG on PI

**Figure 1:**
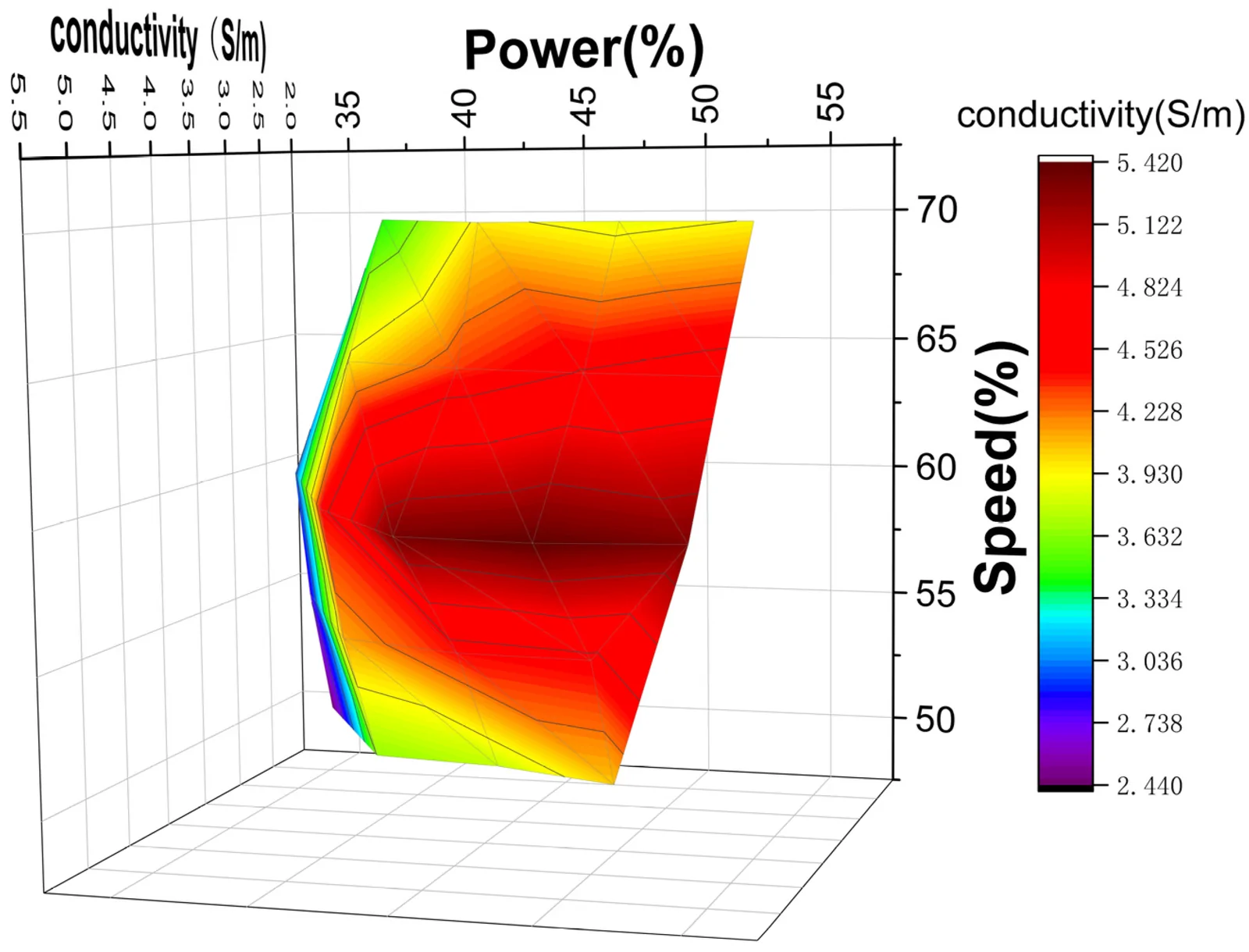
Three-dimensional view of the conductivity of LIG under different laser parameters.

To improve the mechanical and electrical properties of LIG, laser power and engraving speed are optimized during the fabrication process. The laser engraving machine used in this study has a maximum power of 3000 mW and a maximum engraving speed of 3 mm/s. A piece of PI film served as the standard template for LIG formation. During the laser treatment process, insufficient heat caused by a very low laser power or high engraving speed could not break and reorganize the chemical bonds in the polyimide (PI) film to form graphene. Conversely, when the laser power is too high or the engraving speed too low, the laser engraving machine might burn through the PI film [51,52]. Hence, the laser scanning speed and power were set within the ranges of 35–55% and 50–70%, respectively. The electrical conductivity of the graphene sheets was evaluated using the four-probe method [53]. From the correlation between conductivity, laser power, and speed (as shown in Table 1) and the corresponding three-dimensional plot (Figure 1), it can be observed that, under a constant scanning speed, the resistance of the LIG electrode decreases with increasing laser power. It is noteworthy that when the scanning speed is set to 35% or 40%, the conductivity of the resulting LIG remains low, regardless of the power variations, which are represented by colors predominantly in yellow or green in the 3D plot. When the power is in the range of 45–50%, the conductivity increases with the rising scanning speed, as reflected in the 3D plot by a gradual color change from yellow-green to deep red. However, when the scanning speed exceeds 60%, the conductivity begins to decrease as the speed increases, and the 3D plot shows a color shift from red to yellow. This indicates that a scanning speed of 60% is optimal within the 45–50% power range. Additionally, it is essential to note that as an electrode material, LIG must possess adequate mechanical strength. During the experiments, it was observed that LIG fabricated with high power tends to delaminate after bending, suggesting insufficient mechanical integrity and rendering it unsuitable for flexible glucose sensor applications. Therefore, a laser power of 45% and a scanning speed of 60% were determined to be the optimal parameters for fabricating LIG electrodes.

### 2.2. Fabrication and Structure Characterizations of NiCo-LDH/LIG

**Figure 2:**
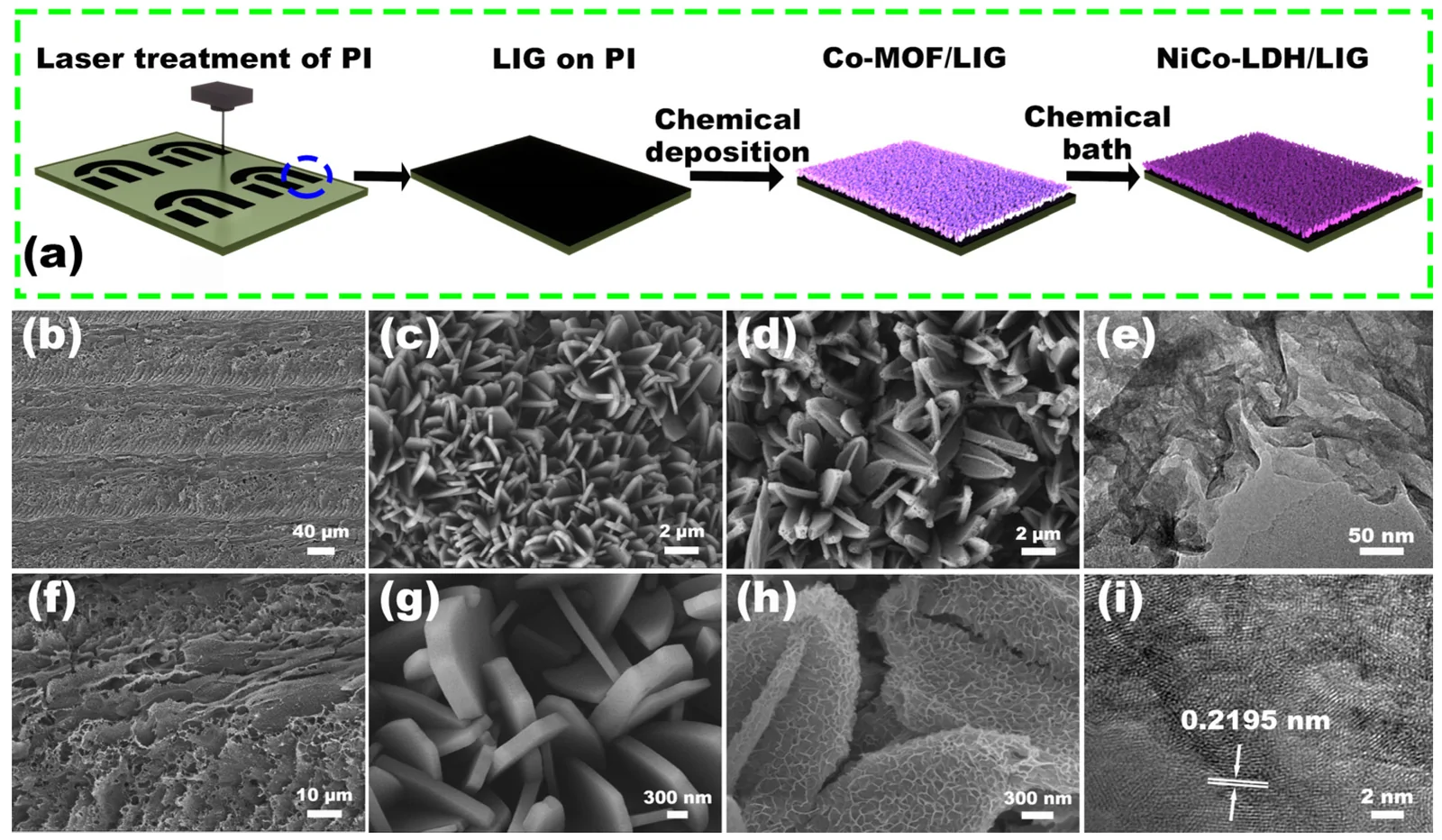
(**a**) NiCo-LDH/LIG preparation process diagram. Low- and high-resolution SEM images of the (**b**,**f**) LIG, (**c**,**g**) Co-MOF/LIG, (**d**,**h**) and NiCo-LDH/LIG. (**e**,**i**) Low- and high-resolution TEM images of the NiCo-LDH.

The construction procedure of the NiCo-LDH/LIG composite electrode produced from MOF appears in Figure 2a. With the optimal laser parameters, the graphene was initially formed on the PI substrate to develop a uniform free-standing electrode (Figure 2b). With the laser ablation, the obtained LIG exhibits porous multilayer graphene walls with abundant micrometric holes and rich edges (Figure 2f). The layered porous structure can provide a large surface area and abundant attachment sites for the in situ growth of ZIF-67. After soaking the LIG electrode in a mixed solution of cobalt ions and 2-methylimidazole, interconnected ZIF-67 nanosheet (polyhedrons) arrays with smooth surfaces are observed to grow on the LIG’s surface (Figure 2c,g), and the average width is about 900 nm. Finally, using a nickel nitrate ethanol solution as the etching agent, Ni ions are introduced into ZIF-67, leading to the release of Co^2^⁺ ions. The subsequent reaction between Co^2^⁺/Co^3^⁺ and Ni ions with hydroxide ions results in the formation of NiCo-LDH. As depicted in Figure 2d, the sheet-like structure of ZIF-67 remained well preserved after the etching process, with the formation of distinct wrinkled structures on the surface of the nanosheets (Figure 2h). This is due to the etching effect of the nickel ions during the soaking and conversion process, leading to the formation of wrinkles and indentations on the surface. This layered structure has the potential to significantly shorten the ion diffusion path, thereby improving conductivity and electrochemical performance. The morphology and microstructure were further investigated by transmission electron microscopy (TEM). Consistent with the SEM results, the edge of NiCo-LDH displays a wrinkled structure (Figure 2e), and the lattice D-spacing of 0.2195 nm could be ascribed to the (015) crystal plane of NiCo-LDH, which corresponds with the XRD investigations.

**Figure 3:**
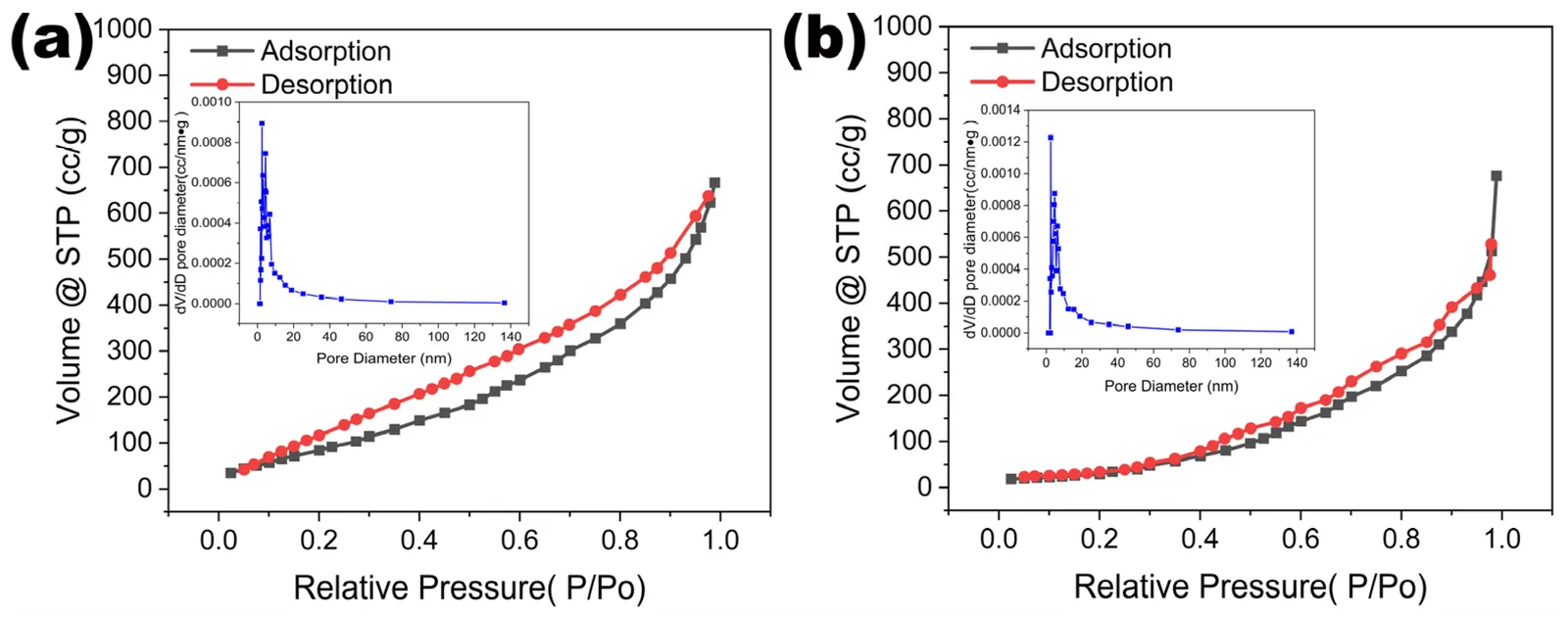
(**a**) LIG’s adsorption/desorption isotherm and pore size distribution. (**b**) NiCo-LDH/LIG’s adsorption/desorption isotherm and pore size distribution.

The BET quantitative analysis method was used to evaluate the specific surface area and pore size distribution of the LIG and NiCo-LDH/LIG samples. The results indicate that these parameters play a crucial role in determining the electrochemical performance. Figure 3a,b illustrate the nitrogen adsorption/desorption isotherms and pore size distribution curves, respectively. According to the measurements, the specific surface area and average pore size of the LIG were 353.88 m^2^/g and 1.048 nm, respectively, while those of the NiCo-LDH/LIG were 263.31 m^2^/g and 2.29 nm. The reduction in the specific surface area of NiCo-LDH/LIG is primarily attributed to the growth of NiCo-LDH within the pores of LIG, which partially blocks the original pore structures, thereby reducing the effective pore volume and, consequently, the specific surface area. Although the specific surface area of NiCo-LDH/LIG is reduced compared to LIG, it still retains a relatively large surface area.

**Figure 4:**
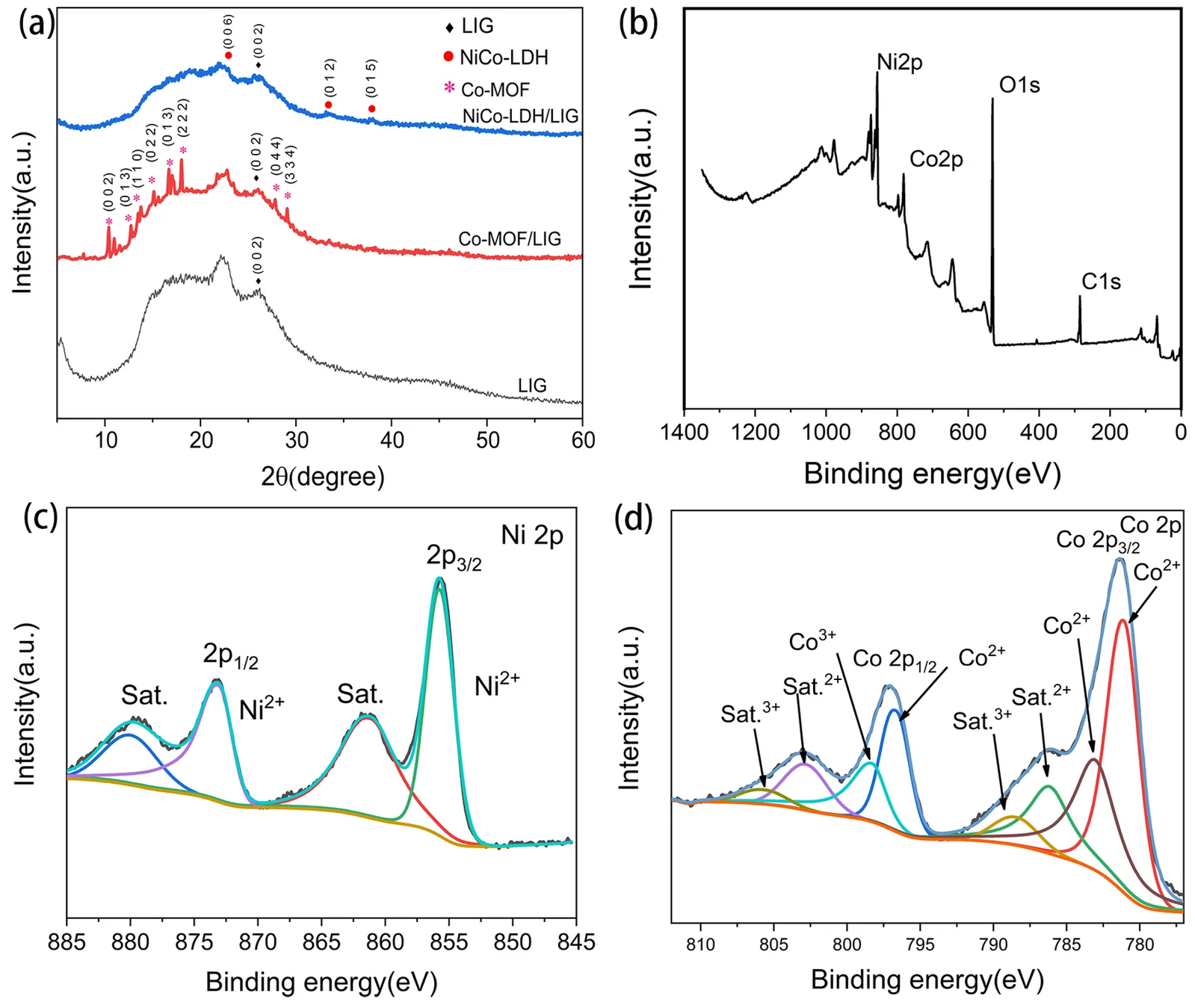
(**a**) XRD patterns of the LIG, Co-MOF/LIG, and NiCo-LDH/LIG. XPS spectra of the NiCo-LDH in the (**b**) survey spectrum, (**c**) Ni 2p, and (**d**) Co 2p.

The complex lattice arrangement and crystal structures of LIG, Co-MOF/LIG, and NiCo-LDH/LIG were characterized using X-ray diffraction (XRD) experimentation (Figure 4a). Peak deconvolution of the LIG shows two broad peaks at 26.38 and 45.22, which could be ascribed to the (002) and (100) planes of graphene [23,54]. As the laser-induced graphene is fabricated on a PI film, the PI substrate exerts influence during the XRD characterization. The broad polymer peak observed at 22.4° and the diffuse peaks between 9° and 33° can be attributed to the contributions of the PI film [55]. As shown in Figure 4a (red line), distinct peaks are observed at 2θ = 10.3°, 12.7°, 13.8°, 15.1°, 16.4°, 17.8°, 27.8°, and 29.1°, corresponding to the (002), (013), (110), (022), (013), (222), (044), and (334) planes of Co-MOF, respectively [43,56]. As the Co-MOF/LIG is immersed in an ethanol solution of nickel nitrate, it gradually transforms into NiCo-LDH/LIG. As shown by the blue line in Figure 4a, the diffraction peaks of Co-MOF have completely disappeared. NiCo-LDH/LIG exhibits three distinct diffraction peaks at 23.02°, 34.38°, and 37.68°, corresponding to the (006), (012), and (015) planes of NiCo-LDH (PDF: 33-0429), indicating the successful formation of MOF-derived NiCo-LDH/LIG composites [57,58].

An analysis of the elemental composition and chemical valence states of the NiCo-LDH/LIG was conducted using X-ray photoelectron spectroscopy (XPS). The survey spectrum, shown in Figure 4b, unequivocally indicated the existence of Ni, Co, C, and O elements. Within the Ni 2p spectrum (Figure 4c), binding energies of Ni 2p^3/2^ and Ni 2p^1/2^ at 856.1 and 873.7 eV, respectively, indicate the presence of the Ni^2+^ valence state [40]. Figure 4d shows two distinct doublets: Co 2p^3/2^ at 781.25 eV and Co 2p^1/2^ at 796.59 eV. Furthermore, the Co^2+^ and Co^3+^ ions exhibit two clearly different oxidation states, as indicated by the difference in energy levels between Co 2p^3/2^ and Co 2p^1/2^, which surpasses 15 eV [59,60]. Two strong satellite peaks can be observed at 786.2 eV and 802.5 eV, which are attributed to Co^2+^. Weak satellite peaks accompanying the strong ones are attributed to Co^3+^ [61,62]. These results further confirm the simultaneous presence of distinct valence states of nickel and cobalt in NiCo-LDH.

### 2.3. Electrochemical Measurements of the NiCo-LDH/LIG Electrode

**Figure 5:**
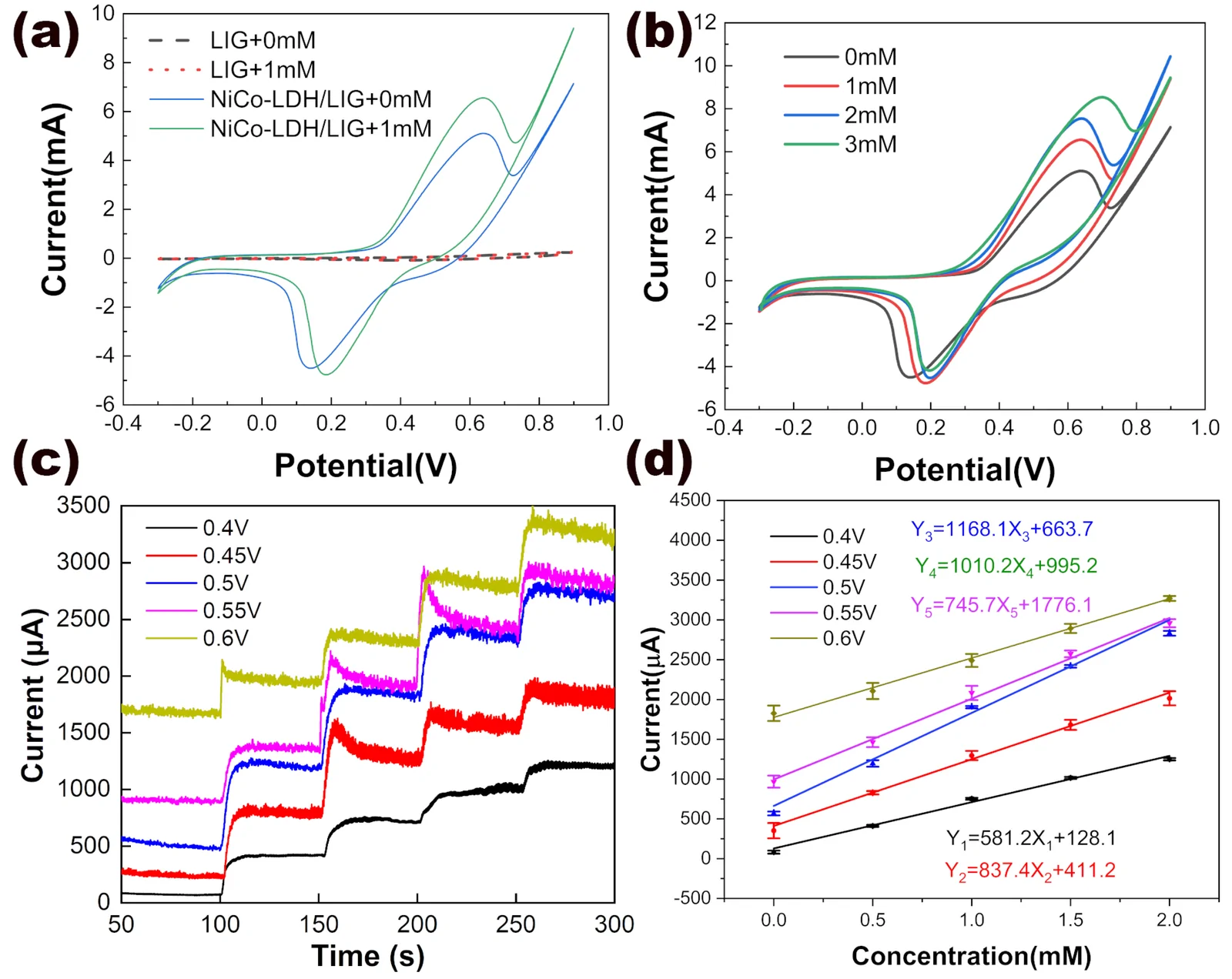
(**a**) CV curves for LIG and NiCo−LDH/LIG in 0.1 M NaOH, with and without 1 mM glucose, were recorded at a scan rate of 10 mV/s. (**b**) CV curves of NiCo−LDH/LIG were obtained in solutions with 0, 1, 2, and 3 mM glucose at a scan rate of 10 mV/s. (**c**) Current responses of five successive injections of 500 μM glucose at different applied voltages. (**d**) The linear fitting results with error bars.

Figure 5a illustrates the cyclic voltammetry (CV) curves for the LIG and NiCo-LDH/LIG in a 0.1 M NaOH solution, both in the presence and absence of 1 mM glucose. The measurements were conducted at a scan rate of 10 mV/s. In both the presence and absence of 1 mM glucose, the CV curves for the LIG electrode exhibit complete overlap and lack any redox peaks. This observation indicates that LIG acts solely as a conductive substrate and does not participate in redox processes. In contrast, the CV analysis of the NiCo-LDH without 1 mM glucose reveals distinct redox peaks. Upon the addition of 1 mM glucose, there is a noticeable increase in the oxidation peak current, suggesting an enhanced reaction intensity. To assess the impact of the glucose concentration on the redox reaction, additional repeated CV experiments were performed at 1 mM, 2 mM, and 3 mM glucose concentrations. For the current peak shifts of 0 mM, 1 mM, 2 mM, and 3 mM, the uncertainties are 0.013 mA, 0.017 mA, 0.014 mA, and 0.011 mA, respectively, while the current peak shifts range from 0.8 mA to 1.2 mA. Since the shift values are significantly larger than the uncertainties, experimental error can be ruled out as the cause. Instead, the shift in the current peaks is attributed to the addition of glucose.

Figure 5b shows that the oxidation peak current between 0.5 V and 0.7 V increases with higher glucose concentrations, indicating significant catalytic activity of NiCo-LDH toward glucose. The reaction catalyzed by NiCo-LDH and glucose is as follows [63]:

(1) $$Ni(OH{)}_{2}+OH^{-}\to NiOOH+H_{2}O+e^{-}$$

(2) $$Co(OH{)}_{2}+OH^{-}\to CoOOH+H_{2}O+e^{-}$$

(3) $$NiOOH+Glucose+e^{-}\to Ni(OH{)}_{2}+Glucolactone$$

(4) $$CoOOH+Glucose+e^{-}\to Co(OH{)}_{2}+Glucolactone$$

To determine the optimal operating voltage of the NiCo-LDH/LIG electrode, 500 μM glucose was added four times to a 0.1 M NaOH solution, and the I–t curves of the NiCo-LDH/LIG electrode were recorded at the applied voltages of 0.4 V, 0.45 V, 0.5 V, 0.55 V, and 0.6 V. The experiment was repeated three times to produce fitted curves with error bars. As shown in Figure 5c, the current in the I–t response curves increases incrementally with the successive addition of 500 μM glucose at all applied voltages, demonstrating the electrode’s reliable performance in glucose detection. According to Figure 5d, the maximum standard deviation during glucose addition was 97 μA, with a maximum relative standard deviation of 7.7%, indicating that the NiCo-LDH/LIG glucose sensor exhibits excellent repeatability and reliability. Moreover, the fitted I–t response curves for each operating voltage in Figure 5d reveal that the curve at 0.5 V exhibits the steepest slope, signifying the highest sensitivity. Therefore, 0.5 V was identified as the optimal operating voltage for the NiCo-LDH/LIG electrode.

To investigate the performance of NiCo-LDH and LIG in comparison to traditional MOF structures and metallic material substrates, comparative experiments were conducted with NiCo-LDH/LIG, NiCo-LDH/Ag, Co-MOF/LIG, and Co-MOF/Ag. The Ag electrodes were fabricated using an inkjet printer (DB-100) from Shanghai Mifang Company, along with conductive silver ink (Base-12). Co-MOF/Ag was obtained by immersing the Ag electrode in a ZIF-67 solution for 2 h, while NiCo-LDH/Ag was prepared by soaking the electrode in an ethanol solution containing nickel nitrate. The preparation of the ZIF-67 solution and the nickel nitrate ethanol solution followed the procedure for NiCo-LDH/LIG fabrication.

**Figure 6:**
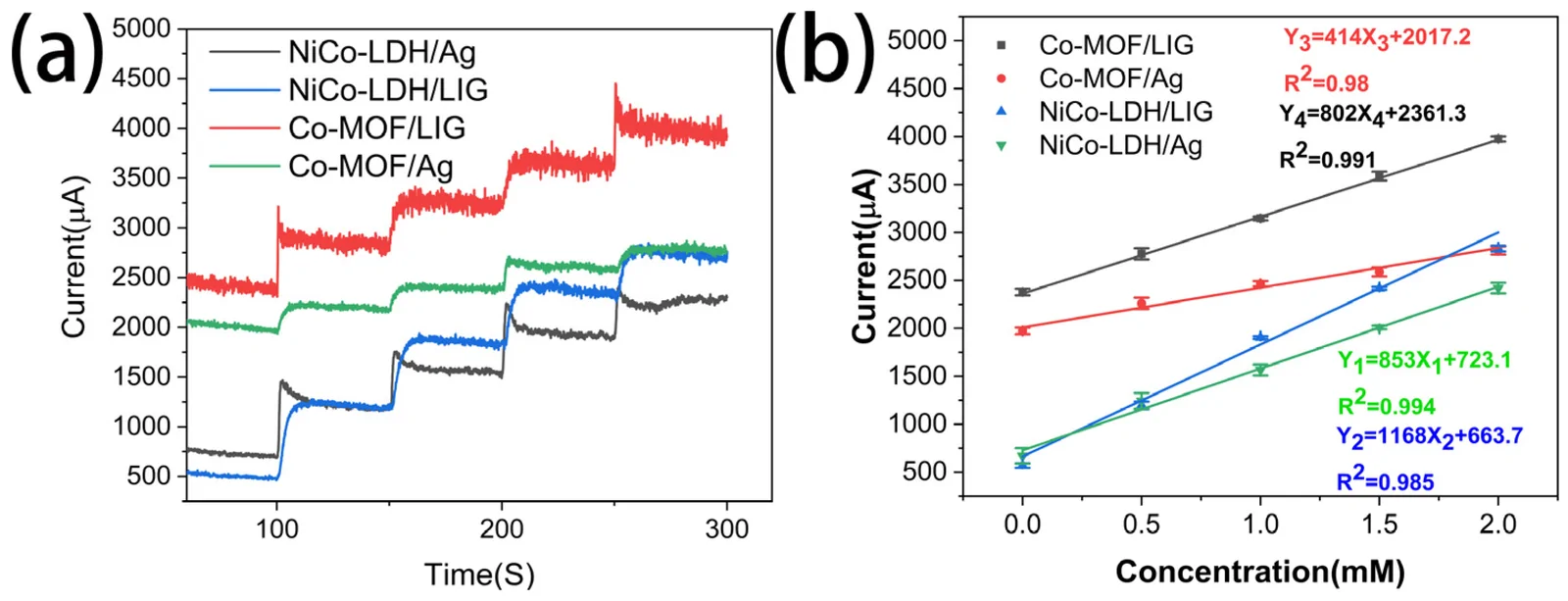
(**a**) Comparative glucose titration experiments of NiCo-LDH/LIG, NiCo-LDH/Ag, Co-MOF/LIG, and Co-MOF/Ag at 0.5 V in 0.1 M NaOH solution. (**b**) Fitting curves with error bars for the comparative experiments.

At an applied voltage of 0.5 V, 500 μM glucose was added in four increments to a 0.1 M NaOH solution, and the corresponding current changes were monitored, as shown in Figure 6a. Each electrode was tested in triplicate, and the results were fitted to generate curves with error bars, as presented in Figure 6b. The maximum standard deviation was 82 μA, and the maximum relative standard deviation was 6.6%, indicating that the test results exhibit excellent reliability and reproducibility. From the fitted curves in Figure 6b, it is evident that regardless of the substrate material (Ag or LIG), electrodes employing NiCo-LDH as the active material demonstrate higher sensitivity compared to those using Co-MOF, indicating the superior glucose detection capability of NiCo-LDH. Furthermore, when the active material remains the same (NiCo-LDH or Co-MOF), LIG-based electrodes exhibit greater sensitivity than Ag-based electrodes. This suggests that LIG provides more active sites for NiCo-LDH and Co-MOF, thereby significantly enhancing the sensitivity of the sensor.

**Figure 7:**
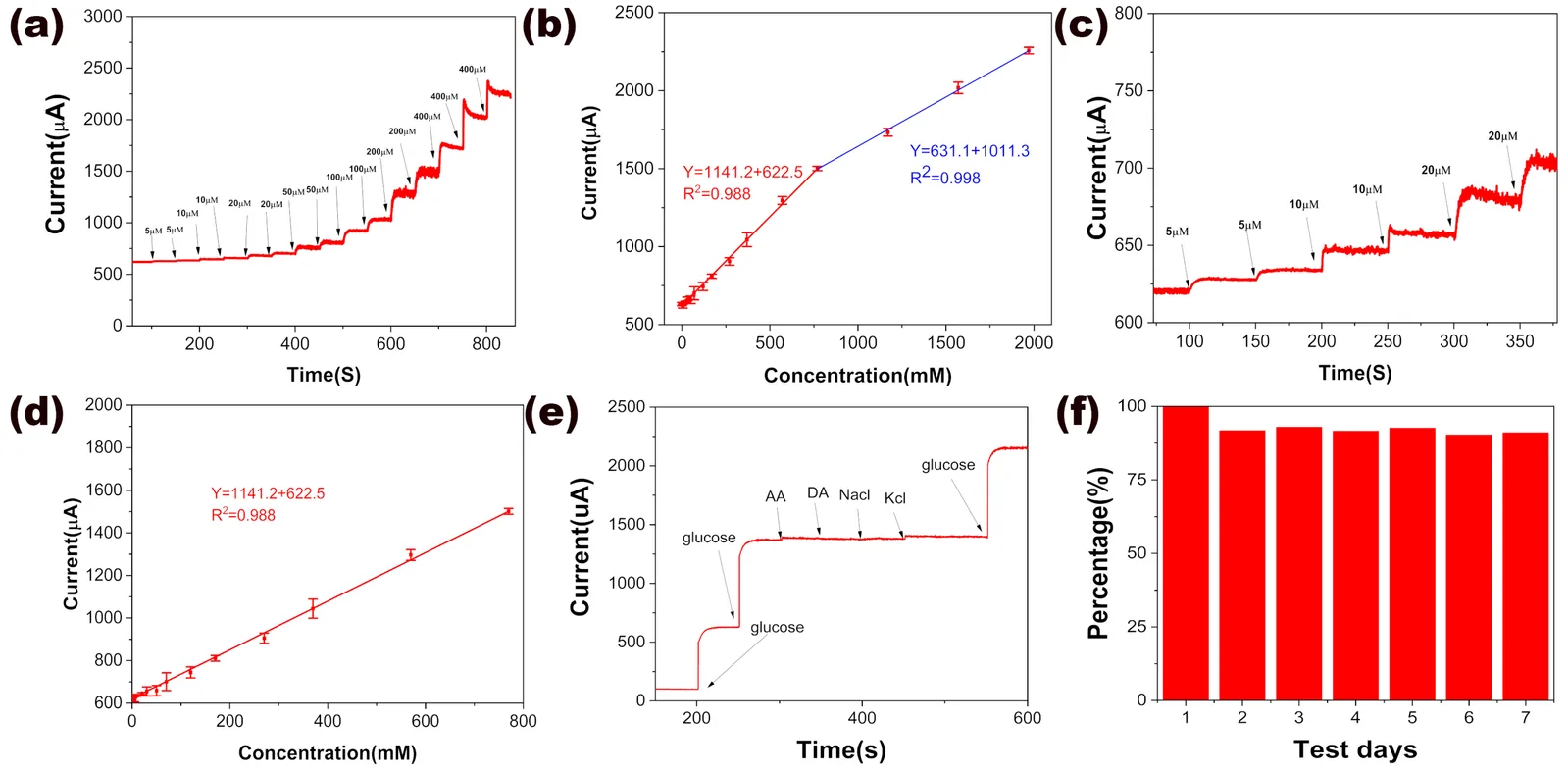
(**a**) A series of amperometric I–t curves were recorded by sequentially adding different glucose concentrations (5, 10, 20, 50, 100, 200, and 400 μM, with each concentration tested twice) to a solution at an applied potential of 0.5 V using Ag/AgCl as the RE. (**b**) Linear fitting curve of the response current with glucose concentration, including error bars. (**c**) Low concentration enlargement of (**a**). (**d**) Low concentration fitting curve in (**b**). (**e**) Current responses of NiCo-LDH/LIG upon the addition of various different interferences. (**f**) Reliability test of NiCo-LDH/LIG over 7 days.

To systematically evaluate the electrochemical performance of the NiCo-LDH/LIG glucose sensor electrode, glucose at different concentrations (ranging from 5 μM to 400 μM) was sequentially added to a 0.1 M NaOH solution under the optimal working voltage of 0.5 V, and the I–t curves were recorded, as shown in Figure 7a. The experiment was repeated three times, generating the graph with error bars shown in Figure 7b, with a maximum standard deviation of 42 μA and a maximum relative standard deviation of 4.3%. Furthermore, to verify whether the differences in the response signals were caused by errors, a t-test was performed on the data within the low-concentration range. The results showed that the signal differences between the points were statistically significant (*p* < 0.05), further confirming the reliability and high sensitivity of the sensor under low-concentration conditions. in Figure 7c, it is also evident that when 5 μM glucose was added, the current response increased significantly, while the current curve showed almost no change when no glucose was added. This further supports the reliability and high sensitivity of the sensor in the low-concentration range.

To quantitatively assess the correlation between the glucose concentration and current response, a linear regression analysis was performed on the amperometric I–t data, with the fitting results shown in Figure 7b. The linear regression equations for the ranges of 5–770 μM and 770–1970 μM were Y = 1141.2X + 622.5 (R^2^ = 0.988) and Y = 631.1X + 1011.3 (R^2^ = 0.998), respectively. The sensitivity of the NiCo-LDH/LIG electrode was calculated to be as high as 1141.2 and 631.1 μA mM⁻^2^ cm⁻^2^. Additionally, the electrode exhibited a low detection limit of 0.437 μM. These findings demonstrate that the NiCo-LDH/LIG electrode possesses excellent sensitivity and a broad linear range, making it highly suitable for glucose detection and providing strong support for the development of biosensors.

It is well known that blood contains not only glucose but also a variety of other biochemical substances and ions that can potentially interfere with glucose detection. While non-enzymatic glucose sensors generally exhibit lower selectivity compared to their enzymatic counterparts, our NiCo-LDH/LIG glucose sensor demonstrates exceptional selectivity. In our study, the NiCo-LDH/LIG glucose sensor showed a significantly high response to 0.2 mM glucose while exhibiting a minimal or nearly no response to common interfering substances present in sweat (Figure 7e). These substances include 0.1 mM NaCl, KCl, uric acid (UA), ascorbic acid (AA), and 1 μM dopamine (DA). This remarkable selectivity demonstrates the sensor’s ability to distinguish glucose from other potentially interfering substances. The exceptional selectivity of the NiCo-LDH/LIG glucose sensor ensures high reliability in practical applications, particularly when analyzing complex biological samples. This characteristic allows the sensor to accurately measure glucose concentrations without significant interference from other substances. Under complicated and tough situations, the NiCo-LDH/LIG glucose sensor can offer precise and dependable glucose measurements. The NiCo-LDH/LIG electrode underwent a seven-day glucose titration test, with measurements taken three times daily. To examine the electrode’s long-term stability, the sensitivity measured on the first day was used as a baseline and compared with the sensitivities measured over the following six days. According to the results shown in Figure 7f, the sensitivity of the NiCo-LDH/LIG electrode on the seventh day was 91.02% of the sensitivity on the first day, indicating only an 8.98% decrease. This result demonstrates that the NiCo-LDH/LIG electrode can maintain excellent performance over extended periods of use, reflecting its outstanding stability.

A comparison of NiCo-LDH/LIG with other non-enzymatic glucose sensors tested in NaOH solution is presented in Table 2. Notably, the NiCo-LDH/LIG sensor demonstrates significantly higher sensitivity and lower detection limits compared to previously reported glucose sensors. The detection limit is calculated using the formula LOD = 3σ/s, where σ represents the standard deviation of the baseline noise, and s denotes the slope of the calibration curve [63]. This exceptional performance can be primarily attributed to the unique electrocatalytic properties of NiCo-LDH, which play a critical role in the effective detection of glucose. Additionally, the synergistic interaction between the LIG substrate and the NiCo-LDH electrocatalyst further enhances the observed electrocatalytic activity.

### 2.4. Electrochemical Measurements of the NiCo-LDH/LIG-Based Glucose Sensor

**Figure 8:**
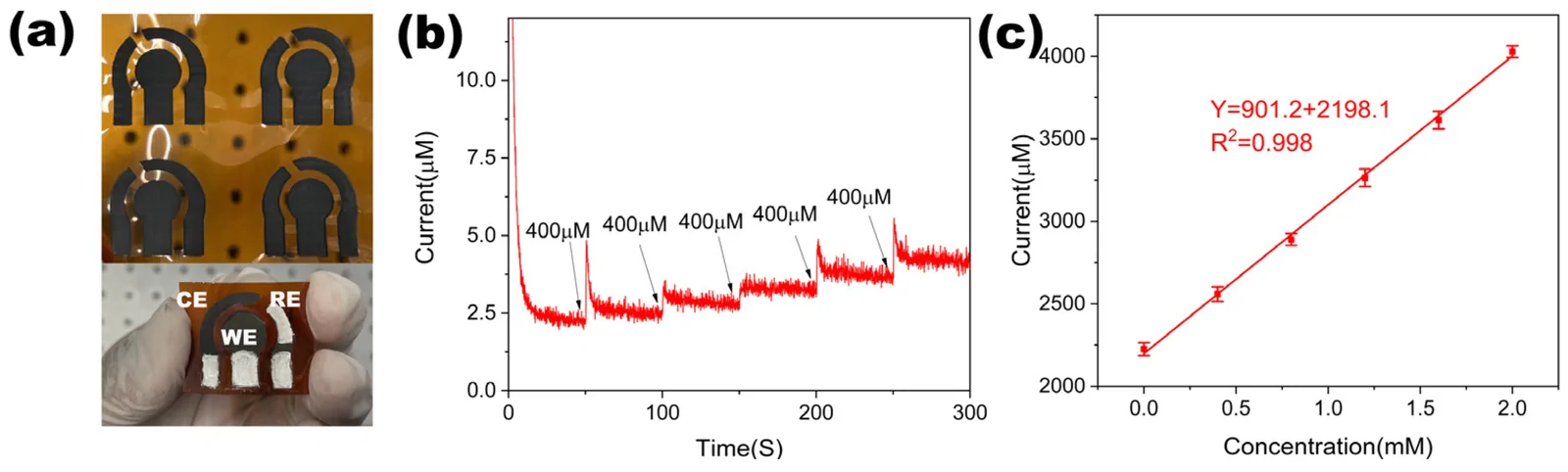
(**a**) Photograph of LIG patterns on PI and the integrated three-electrode glucose sensor device. (**b**) Amperometric I–t curves of successive additions of the same concentrations of glucose (400 μM) at an applied potential of 0.5 V. (**c**) Linear fitting curve of response current with glucose concentration.

To further explore the practical applications of NiCo-LDH/LIG, an integrated three-electrode sensor was assembled with NiCo-LDH/LIG as the working electrode (WE), Ag/AgCl/LIG as the reference electrode (RE), and bare LIG as the counter electrode (CE), as shown in Figure 8a. The glucose sensor device was placed in a 0.1 M NaOH solution, and 400 μM glucose was continuously added, with the experiment repeated three times. The resulting I–t curve for the continuous addition of glucose is recorded in Figure 8b. Each glucose addition produces a clear and consistent stepwise increase in current, indicating a reliable current response of the integrated sensor to glucose. Figure 8c shows the correlation between the glucose concentration and response current with error bars. A strong linear correlation between the current response and glucose concentration is evident. The linear regression equation is Y = 0.901X + 2198.1, with a regression coefficient R^2^ of 0.998. This indicates that the integrated glucose sensor device exhibits a strong linear response and high sensitivity.

**Figure 9:**
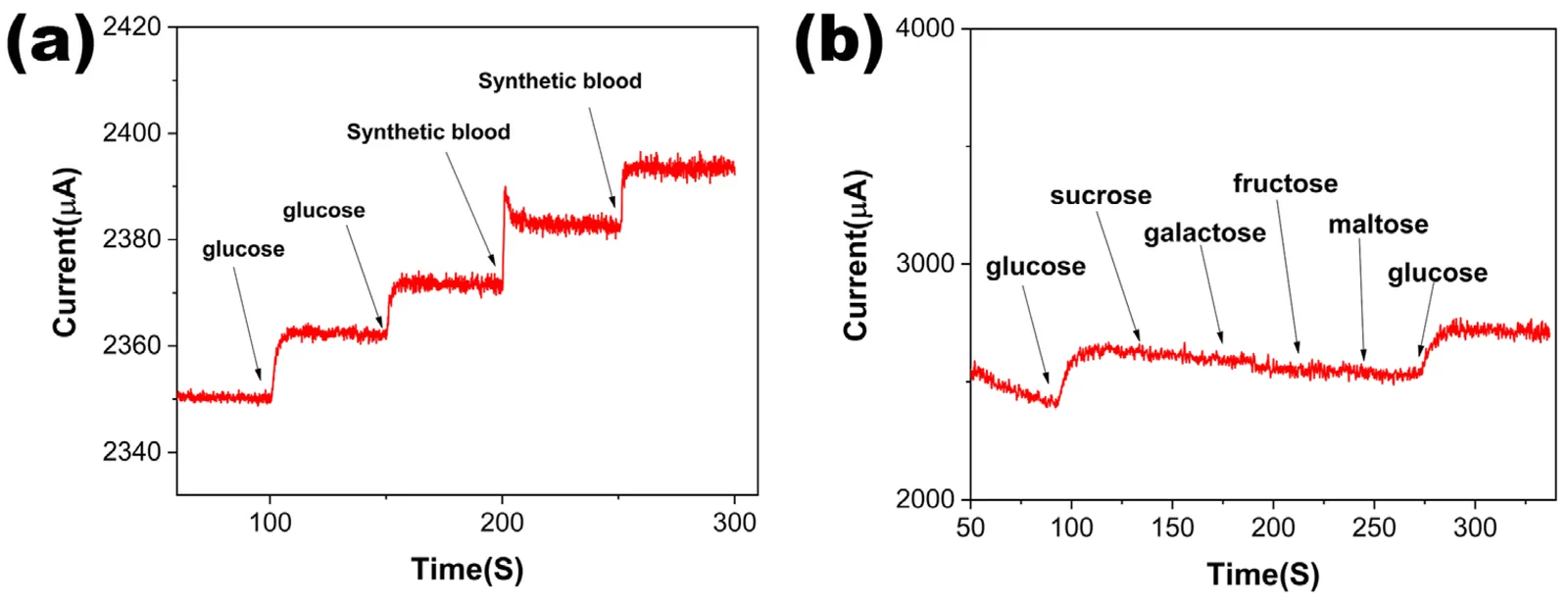
(**a**) NiCo-LDH/LIG sensor was tested with synthetic blood and glucose solution in 0.1 M NaOH solution. (**b**) Comparison test of glucose with other sugars in 0.1 M NaOH solution.

To evaluate the practical performance of NiCo-LDH/LIG in real-world detection, synthetic blood (purchased from Fuzhou Feijing Biotechnology Co., Ltd.) was used to simulate a blood environment with a glucose concentration of 6 mM (the normal human blood glucose concentration range is 3.9–7.8 mM). For further experiments, a 6 mM glucose standard solution was prepared. Subsequently, 50 μL of glucose standard solution and synthetic blood were, respectively, added dropwise into 20 mL of 0.1 M NaOH solution, with the experimental results shown in Figure 9a. Based on the measurements, the sensor’s recovery rate was calculated to be 97.3% to 101.7%, with a relative standard deviation (RSD, N = 5) of 2.1%, indicating that the NiCo-LDH/LIG glucose sensor demonstrates excellent stability and accuracy in practical applications.

Additionally, to further investigate whether other common sugars interfere with the NiCo-LDH/LIG glucose sensor, 0.2 M solutions of glucose, sucrose, galactose, fructose, and maltose were added dropwise to a 0.1 M NaOH solution. The results, as shown in Figure 9b, indicate a significant current response upon the addition of glucose, while the addition of other sugars did not cause any noticeable current changes. This further confirms that the NiCo-LDH/LIG glucose sensor has excellent selectivity and is not affected by common sugars.

To comprehensively investigate the outstanding electrochemical performance of the NiCo-LDH/LIG glucose sensor, the contribution of LIG is particularly critical and is reflected in the following aspects:

Firstly, LIG provides an excellent structural foundation for the entire sensor. The surface of LIG is rich in wrinkles and pores (as shown in Figure 2b,f), significantly increasing the specific surface area and active sites. This provides abundant attachment points for the in situ growth of Co-MOF and the in situ conversion of NiCo-LDH, thereby increasing the loading of NiCo-LDH per unit area. As a result, the glucose sensor achieves enhanced sensitivity.

Secondly, as a conductive framework, LIG imparts outstanding electrical conductivity to the sensor. The excellent conductivity of LIG, combined with the high electrochemical activity of NiCo-LDH, results in a strong synergistic effect that significantly enhances the sensitivity of the sensor.

Thirdly, the three-dimensional porous network structure of LIG further accelerates ion diffusion and enhances the reaction rate. Compared to other similar systems, the three-dimensional support provided by LIG not only improves the performance of the nanomaterials but also leads to superior glucose detection performance.

Additionally, the uniform modification of LIG with NiCo-LDH nanostructures significantly improves the mechanical and electrochemical stability of the composite structure, making it well-suited for long-term use in flexible and wearable devices, which is crucial for practical applications in health monitoring technologies.

Finally, from an economic perspective, LIG technology offers significant advantages over traditional methods such as screen printing and inkjet printing. The production process for LIG is generally simpler and more efficient, enabling the rapid fabrication of large-area electrodes. Furthermore, the material costs associated with LIG technology are relatively low, enhancing its feasibility and competitiveness for large-scale production. The high controllability and repeatability of laser processing also help reduce material waste during production, further lowering manufacturing costs.

## 3. Materials and Methods

### 3.1. Materials Preparation

Co(NO_3_)_2_·6H2O, Ni(NO_3_)_2_·6H_2_O, NaCl, KCl, DA, UA, AA, sucrose, galactose, maltose, and fructose were purchased from Sinopharm Chemical Reagent Co., Shanghai, China. Both 2-Methylimidazole and anhydrous ethanol come from Shanghai Titan Technology Co., LTD., Shanghai, China. All chemicals were analytically pure and were not further processed. The synthetic blood was purchased from Fuzhou Feijing Biotechnology Co., Ltd. (Fuzhou, China) for research.

### 3.2. Synthesis of NiCo-LDH Sensing Electrode

A DAJA-DJ6 laser engraving machine (Dongguan DAJA, Dongguan, China) was used for the material synthesis, which includes the following steps. First, a 100 μm thick PI film (Suzhou Meikesi Plastic Products Co., Ltd., Suzhou, China) was cut into 3 cm × 3 cm sheets. The PI film was then ultrasonically cleaned with anhydrous ethanol and deionized water, followed by drying in air. The prepared PI film was firmly fixed around the edges and laid flat under the focal point of the laser engraving machine. A design pattern was created for the engraving process, and the parameters of the laser engraving machine were adjusted as follows: laser power at 45%, scan rate at 60%, and contrast at 50%. The machine parameters were adjusted, and the film was engraved. After the engraving process was completed, the film was rinsed with deionized water and anhydrous ethanol and then dried to obtain a laser-induced graphene electrode. Subsequently, 1.642 g of dimethylimidazole and 0.7276 g of cobalt nitrate hexahydrate were dissolved in 50 mL of deionized water. After stirring for 10 min, the dimethylimidazole solution was mixed with the cobalt nitrate solution and stirred for 5 min to prepare the ZIF-67 solution. Next, the LIG electrode was submerged vertically in the ZIF-67 solution for a duration of 2 h. After thorough rinsing with deionized water, the Co-MOF/LIG electrode was dried at 50 °C for 5 h. The Co-MOF/LIG electrode was soaked in a 10 mM solution of nickel nitrate ethanol over 8 h, resulting in the conversion of Co-MOF/LIG into NiCo-LDH/LIG. After immersion, the electrode was thoroughly rinsed with deionized water and dried in an oven at 50 °C for approximately 5 h to obtain the NiCo-LDH/LIG electrode.

### 3.3. Fabrication of NiCo-LDH/LIG-Based Glucose Sensor

The glucose sensor was constructed using a three-electrode framework consisting of NiCo-LDH/LIG as the WE, LIG as the CE, and Ag/AgCl/LIG as the RE. Firstly, the CE, WE and RE were made, respectively, by using the three rectangular LIG electrodes induced by one sheet of PI film. Secondly, the LIGs for the CE and RE were covered with PET film, leaving the LIG part for the WE exposed. Following the completion of the covering, the PI film was used to create the NiCo-LDH/LIG WE. The PET film was removed after NiCo-LDH/LIG was created in order to rinse and dry. The LIG electrode was covered with Ag/AgCl paste to create the Ag/Agcl/LIG RE after waiting for the drying to be finished, and it was then placed into the drying oven to dry to create the NiCo-LDH/LIG glucose sensor.

### 3.4. Material Characterizations

The surface morphology of the samples was characterized using field-emission scanning electron microscopy (FESEM, NovaNano-450 FEI, Hillsboro, OR, USA) and transmission electron microscopy (TEM, JEM-2100, JEOL, Tokyo, Japan). The elemental composition and chemical states were analyzed by X-ray photoelectron spectroscopy (XPS, Escalab250, Thermo Fisher Scientific, Waltham, MA, USA), while the crystal structure was determined using X-ray diffraction (XRD, Bruker, Bremen, Germany). The Keithley 2400 SourceMeter (Cleveland, OH, USA) was used to perform four-probe conductivity measurements on the graphene sheets. XPS analysis was performed using Advantage version 5.9931 software, with the binding energy reference set to the C 1s peak at 284.8 eV. The fitting was conducted using a Gaussian–Lorentzian mixed mode with the Powell algorithm, and all other parameters were set to their default values. The specific surface area and pore size distribution were determined using the Autosorb IQ gas adsorption analyzer (Quantachrome Instruments, Boynton Beach, FL, USA), with nitrogen gas used as the adsorbate.

### 3.5. Electrochemical Measurements

The electrochemical performance of the glucose sensor was evaluated using a PARSAT-3000A-DX (AMETEK, Newark, DE, USA) electrochemical workstation with a standard three-electrode configuration. The electrolyte utilized was a 0.1 M NaOH solution, with the NiCo-LDH/LIG electrode functioning as the WE, Pt foil as the CE, and Ag/AgCl as the RE. Glucose detection was evaluated using chronoamperometry with continuous stirring at an ambient temperature, applying an optimal potential of 0.5 V. This work presents an analysis of the resulting current response curve.

## 4. Conclusions

In summary, a novel MOF-derived NiCo-LDH/LIG composite electrode with a large surface area and high conductivity is fabricated by modified laser treatment and a follow-up chemical deposition. The NiCo-LDH/LIG sensor exhibits an ultra-high sensitivity of 1141.2 and 631.1 μA mM^−2^ cm^−2^ within the linear ranges of 0–770 μM and 770–1970 μM, respectively, as well as a low detection limit of 0.437μM. Moreover, the NiCo-LDH/LIG exhibits excellent stability and minimal interference from other substances. Additionally, the sensor demonstrates excellent reproducibility, maintaining 91.02% of its sensitivity after 21 tests over a period of seven days. A flexible three-electrode-integrated LIG-based non-enzymatic glucose sensor was fabricated and showed high sensitivity. The high sensitivity, low detection limit, excellent reproducibility, and strong selectivity make the NiCo-LDH/LIG electrode highly promising in the field of non-enzymatic glucose sensors.

## Figures and Tables

**Table 1:** Conductivity of LIG under different laser parameters.

Power (%)	Speed (%)	Conductivity (S/m)
35	50, 55, 60	2.44, 2.76, 2.95
40	50, 55, 60, 65, 70	3.65, 4.12, 4.42, 3.97, 3.42
45	50, 55, 60, 65, 70	3.76, 4.41, 5.33, 4.34, 3.97
50	50, 55, 60, 65, 70	4.05, 4.46, 5.42, 4.54, 3.86
55	60, 65, 70	5.32, 4.65, 3.94

**Table 2:** Comparison between NiCo-LDH/LIG with other reported glucose sensors.

Materials	Linear Range (mM)	Sensitivity (μA Mm^−1^ cm^−2^)	LOD (μM)	Reference
Cu NPS/LIG	—	495	0.39	[47]
Pt@Pd/PP/LIG	0.01–9.2	247.3	3	[64]
Au/Ni	0–30	30.58	5.84	[65]
Au/Fe_2_O_3_/f-MWCNTs	0.01–0.08	512.4	1.71	[66]
Ag@ZIF-67/GCE	0.002–1	379	0.66	[67]
NiCo-LDH/GNR/GCE	0.005–0.8	344	0.60	[68]
AgNW/NiCo LDH	0.002–6	71.4	0.66	[69]
NiCo-LDH/LIG	0.05–1.97	1141.2	0.43	This work

## Data Availability

The data presented in this study are available in the article and can be shared upon request.
